# Agreement in Subjective Periodontal Findings in Undergraduate Dental Training: A Retrospective Observational Study of Bleeding on Probing and Tooth Mobility

**DOI:** 10.3390/dj14040235

**Published:** 2026-04-14

**Authors:** Patrick Jansen, Felix Krause, Andreas Braun, Ellen E. Jansen

**Affiliations:** Department of Operative Dentistry, Periodontology and Preventive Dentistry, Rheinisch-Westfälische Technische Hochschule (RWTH) University Hospital, 52074 Aachen, Germany

**Keywords:** periodontal diagnosis, dental education, bleeding on probing, tooth mobility, examiner variability, examiner agreement

## Abstract

**Background/Objectives:** Accurate assessment of periodontal parameters such as bleeding on probing (BoP) and tooth mobility is essential for diagnosis and treatment planning. In contrast to metric measures, these findings represent more subjective clinical parameters, potentially increasing examiner-related variability, particularly among less experienced examiners. This study evaluated agreement patterns between student recordings and educator verification recordings and assessed the influence of educational level and anatomical factors. **Methods:** BoP and tooth mobility data recorded by undergraduate dental students (ICC1, ICC4, state examination) and verified by licensed dental educators were retrospectively analyzed, comprising 6504 BoP sites and 1084 mobility recordings. Agreement and directional disagreement (under-/over-rating) were analyzed. Statistical analyses evaluated the effects of training stage, anatomical site, and periodontal severity. Associations with probing depth deviations and patient age were also examined. **Results:** Overall agreement was high for both parameters (BoP: 86%; mobility: 91%). Nevertheless, statistically significant differences were observed across educational levels (global Chi^2^ tests *p* < 0.001). ICC1 students predominantly underestimated findings, whereas ICC4 students more frequently overestimated them; results stabilized in the state examination. Anatomical location significantly influenced disagreement patterns, with anterior and distal sites showing higher variability. BoP deviations were significantly associated with probing depth inaccuracies (r = 0.6; *p* < 0.001) and patient age (r = 0.187; *p* < 0.05). **Conclusions:** Within the limitations of this retrospective study, student recording accuracy for subjective periodontal parameters appears to be influenced by training stage and anatomical site. These findings may highlight the importance of structured calibration and targeted training strategies to improve diagnostic reliability in undergraduate periodontal education.

## 1. Introduction

Accurate clinical assessment is a key competency in undergraduate dental education. In periodontology, clinical findings do not serve documentation alone; they directly influence diagnosis, treatment planning, and supportive periodontal care [1]. In teaching clinics, periodontal charting therefore represents both a diagnostic procedure and a central educational task.

Among routinely recorded periodontal parameters, bleeding on probing (BoP) and tooth mobility deserve special attention. BoP provides information related to inflammatory activity, while mobility reflects functional stability and periodontal support [2,3]. At the same time, both measures are examiner-dependent and prone to variability, which is likely amplified in undergraduate settings where manual skills and clinical judgement are still developing [4,5].

BoP is widely used as a clinical sign of gingival and periodontal inflammation [6]. In daily practice it is recorded as a dichotomous finding (present/absent), typically assessed after probing is performed to determine probing pocket depths (PPDs) within a defined time interval [6,7]. Histologically, bleeding reflects inflammatory changes in the sulcular epithelium and increased vascular permeability, facilitating bleeding upon mechanical stimulation [8]. BoP contributes to periodontal diagnosis and monitoring [6]. However, its diagnostic meaning is asymmetric: the absence of BoP is generally considered a strong indicator of periodontal stability, whereas a positive finding requires careful interpretation due to limited specificity [9]. Recent evidence confirms that bleeding on probing remains a key parameter in periodontal assessment, while also demonstrating that its association with other periodontal parameters and disease progression may be limited [10,11]. Inaccurate BoP recordings may therefore misclassify inflammatory activity and influence clinical decisions such as treatment intensity and recall intervals.

Despite its apparent simplicity, BoP assessment is technique-sensitive. Probing force is a key determinant: even modest differences can affect bleeding outcomes and may lead to false-positive recordings when excessive force is applied [12,13]. Furthermore, anatomical access and local conditions (e.g., deep pockets, interproximal sites, posterior regions) complicate probing angulation and consistency [6]. For students, these practical limitations are particularly relevant and may result in systematic rather than random disagreement patterns. Previous studies have reported relevant inter-examiner variability for BoP, with reproducibility under routine clinical conditions often being only moderate [14,15]. However, calibration and structured training have been shown to improve the consistency of periodontal recordings and examiner agreement [15,16]. Recent evidence further highlights that bleeding on probing is influenced by multiple local and examiner-related factors and may show only limited association with other periodontal parameters or disease progression [17,18].

Tooth mobility is another established periodontal finding. Increased mobility may reflect periodontal breakdown with loss of periodontal support, inflammation of the periodontal ligament, and traumatic occlusal loading [19,20]. In undergraduate teaching clinics, mobility is usually assessed manually (e.g., Miller’s classification) by applying alternating pressure and estimating displacement. This approach is practical but subjective, particularly in borderline cases and when assessing low-grade mobility [19,20]. Although objective devices for mobility quantification have been described and may offer improved reproducibility, they are rarely available in routine undergraduate training [21,22,23]. Consequently, students continue to rely predominantly on manual assessment, and the reliability of student mobility recordings under routine conditions remains an important educational and clinical question.

While the accuracy of student assessments for probing depth (PD) has been investigated previously [24], less is known about the agreement between student and educator recordings for BoP and tooth mobility under routine clinical teaching conditions. In particular, it remains unclear how accuracy differs across educational levels, whether disagreement follows systematic anatomical patterns, and whether it relates to periodontal severity or probing performance.

Therefore, the primary aim of the present study was to assess the agreement and directional disagreement of student recordings for BoP and tooth mobility across different educational stages by comparing student findings with educator verification recordings performed by supervising dentists. The secondary aims were to evaluate whether disagreement patterns were associated with anatomical location (anterior vs. posterior; buccal vs. oral; interproximal vs. mid-sites), periodontal severity, patient age, and deviations in probing depth measurements. The working hypothesis of the present study was that student recordings would show systematic deviations from educator reference recordings, influenced by educational level, anatomical site location, and periodontal disease severity.

## 2. Materials and Methods

In this retrospective study, periodontal records from the Department of Operative Dentistry, Periodontology and Preventive Dentistry at Aachen University Hospital were evaluated. While the educational setting and periodontal curriculum (Aachen Periodontal Therapy Concept, APTC) were identical, the current study specifically analyzed agreement and disagreement patterns in student recordings of bleeding on probing (BoP) and tooth mobility.

A retrospective study design was chosen to analyse the accuracy of student periodontal recordings by using existing clinical documentation containing a large number of measurement points. As students were unaware that their routine recordings would later be analysed, performance alterations due to observation were unlikely. Data collection spanned from January 2021 to April 2024, with each patient measured by a single student. Periodontal findings were documented digitally using the ParoStatus.de software (Parostatus GmbH, Berlin, Germany) and exported in Microsoft Excel (Microsoft Corp., Redmond, WA, USA) for analysis. Over the study period, multiple student cohorts were involved, representing different stages of dental student training. Across the study period, the underlying routine dataset comprised 13,530 BoP site recordings and 2255 mobility recordings obtained during periodontal examinations in the clinical student course. After applying predefined eligibility criteria (see “Section 2.2”), 6504 BoP sites and 1084 mobility recordings remained for the final analysis. These findings were noted during clinical examinations and subsequently checked and documented by licensed dental educators. Verification was performed by multiple licensed supervising dentists involved in undergraduate periodontal teaching as part of routine clinical teaching; due to this workflow, blinding of the examiners to student findings was not feasible. Educator recordings were therefore considered the pragmatic reference standard for the present agreement analysis. This study included students in their clinical study period, from the fourth year of study (first clinical course, integrating the fields of cariology, periodontology, endodontology, restorative and pediatric dentistry [ICC1], seventh semester), the fifth year of study (fourth clinical course, integrating the fields of cariology, periodontology, endodontology, restorative and pediatric dentistry [ICC4], tenth semester), and the state examination (SE, the last examination before licensing).

### 2.1. Periodontal Training Concept

**Figure 1:**
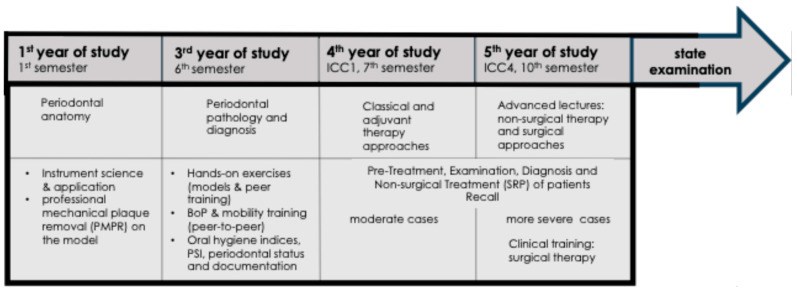
Structure of the periodontal training curriculum within the undergraduate dental program at the Department of Operative Dentistry, Periodontology and Preventive Dentistry at RWTH Aachen University Hospital: Timeline of undergraduate periodontal education, including the introduction of BoP assessment and tooth mobility grading during supervised peer-to-peer training (6th semester), followed by progressively supervised clinical patient care (ICC1: first clinical course; ICC4: advanced clinical course) and the state examination.

Periodontal education is based on the departmental undergraduate training concept, which integrates preclinical simulation training with supervised clinical patient care (Figure 1). Clinical treatment procedures follow the Aachen Periodontal Therapy Concept (APTC). During the clinical courses (ICC1 and ICC4), periodontal diagnostics and therapy are performed under continuous supervision and immediate verification by licensed dental educators, with increasing case complexity.

BoP assessment and tooth mobility grading were taught as part of the structured undergraduate curriculum and routinely performed under supervision during clinical training.

### 2.2. Data Selection

**Figure 2:**
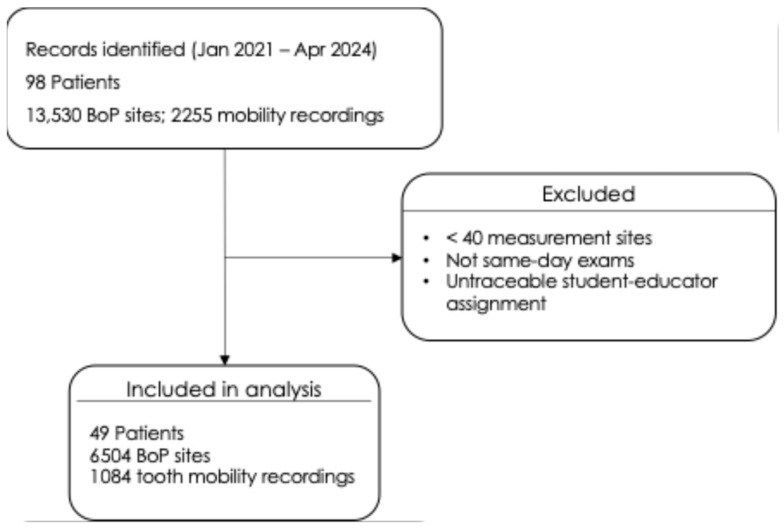
Flow diagram of dataset eligibility and selection for the analysis of bleeding on probing (BoP) and tooth mobility recordings in undergraduate periodontal charting (January 2021–April 2024).

Data sets were included in the evaluation if the patients had at least 40 measuring points, the examinations took place on the same day and the data sets could be clearly assigned to the dentist/student group. After applying these criteria, 49 patient records were eligible for analysis, including 21 from the ICC1 course, 19 from the ICC4 course, and 10 from the state examination (SE). From this eligible sample, 6504 BoP site recordings and 1084 tooth mobility recordings (teeth) were included in the final analysis (Figure 2).

### 2.3. Data Collection

Periodontal charting was performed at six sites per tooth (mesio-buccal, buccal, disto-buccal, mesio-oral, oral, disto-oral). Following gentle periodontal probing to determine probing pocket depths (UNC-15 periodontal probe (Hu-Friedy, Chicago, IL, USA); probing force approximately 0.25 N), BoP was assessed at the same sites after 10–30 s and documented as present or absent [1]. Tooth mobility was assessed manually using an instrument handle by applying alternating pressure and graded according to Miller’s classification: grade 0 (physiological mobility), grade 1 (horizontal displacement up to 1 mm), grade 2 (horizontal displacement 1–2 mm), and grade 3 (vertical mobility and/or movement in response to tongue pressure) [25]. All findings were recorded digitally using ParoStatus.de. After completion of charting by the student, recordings were verified by the supervising licensed dental educator as part of routine clinical teaching. The supervising dentist performed a second periodontal examination, re-assessing bleeding on probing and tooth mobility at all recorded sites. The educator findings were documented separately in the digital periodontal chart and subsequently compared with the student recordings. Measurement divergences were determined based on the differences between student and educator recordings at the site level. No periodontal instrumentation, including plaque removal or subgingival treatment, was performed between the student examination and the supervisor verification. Educator verification was performed by licensed dentists involved in undergraduate periodontal teaching at the department. All supervising dentists were trained staff members of the department with routine clinical experience in periodontal examination and treatment and regular involvement in periodontal teaching activities. In addition, they participated in postgraduate continuing education programs activities and internal calibration sessions within the department. However, no formal calibration procedure was conducted specifically for this study.

### 2.4. Data Analysis

For the following analysis, the data were exported to Microsoft Excel (version 2405; Microsoft Corp., Redmond, WA, USA) and evaluated in Excel and IBM SPSS Statistics (version 30; IBM Corp., Armonk, NY, USA). BoP and tooth mobility represent subjective clinical assessments (binary/ordinal outcomes) and were therefore analysed primarily as directional disagreement (under- vs. over-rating) relative to educator recordings. For each site/record, the difference between student and educator recordings was calculated and summarized as agreement (difference = 0) and directional disagreement (under- and over-rating relative to educator findings). The proportion of agreement and disagreement was calculated overall and stratified by anatomical site location and educational level (ICC1, ICC4, SE). Additionally, disagreement in BoP and mobility recordings was correlated with probing depth accuracy, patient age, and educational level. The significance level was set at *p* < 0.05. Probing depth accuracy was calculated as previously described in our related analysis of the same dataset [24]. In brief, probing depth accuracy was defined based on the deviation between student and educator probing depth measurements at the site level.

The study was conducted in full accordance with local and global ethical guidelines (World Medical Association Declaration of Helsinki, Fortaleza version, 2013) and approved by the local ethics committee (CTC-A-Nr.25-261, EK 25-233, RWTH Aachen University). As this was a retrospective analysis of anonymised routine data, no additional patient consent was required.

## 3. Results

The analysis focused on discrepancies between student and educator recordings of bleeding on probing (BoP) and tooth mobility. Results are reported as overall agreement as well as directional disagreement (under- vs. over-rating relative to educator reference values), including subgroup analyses by educational level, periodontal severity, and anatomical site.

### 3.1. Study Sample

After applying eligibility criteria, the final analytical dataset comprised 49 patients with 6504 BoP site recordings and 1084 tooth mobility recordings available for analysis (Figure 2). The study population consisted of 20 women (40.8%) and 29 men (59.2%), with a mean age of 60.9 years (SD = 13.0; median = 61.0; range: 24.1–82.1 years).

### 3.2. Bleeding on Probing

#### 3.2.1. Distribution of BoP Recordings and Overall Student–Educator Agreement

**Figure 3:**
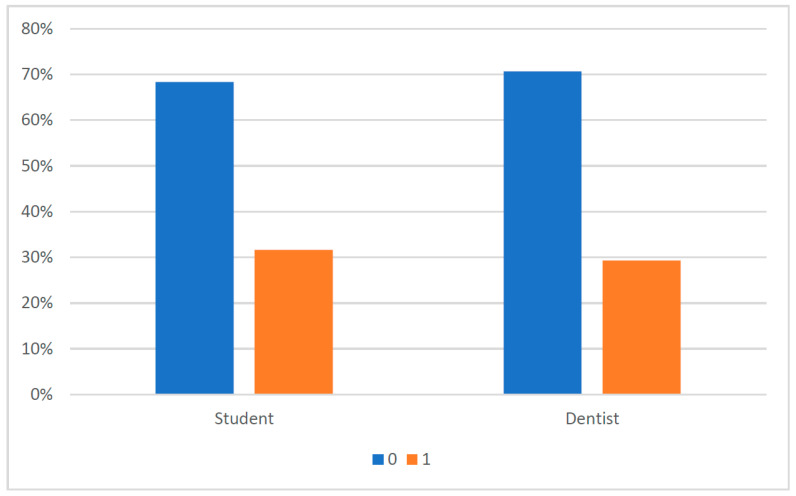
The percentage distribution from no bleeding (0) to detected bleeding (1) is shown, both by the cohort of students and dentists.

**Figure 4:**
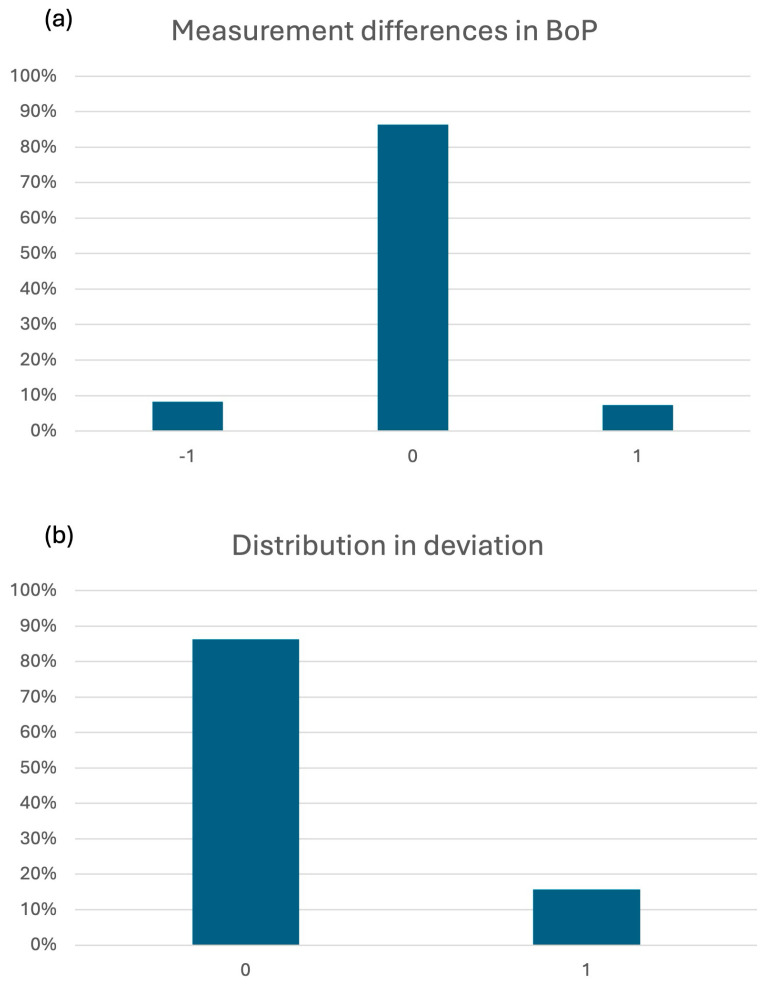
(**a**) Divergences in measurements of bleeding on probing (BoP). Negative values (−1) indicate underestimation by the student relative to the educator recording, 0 indicates agreement, and positive values (+1) indicate overestimation. (**b**) Percentage distribution of the amounts of divergence (no divergence [0] vs. divergence [1]).

The raw distribution of BoP recordings was comparable between students and educators, with similar proportions of BoP-negative and BoP-positive sites (Figure 3). When comparing recordings site-by-site, the majority of sites showed agreement between students and educators. Disagreement occurred in both directions: students either recorded no bleeding where the educator recorded bleeding (under-rating) or recorded bleeding where the educator recorded no bleeding (over-rating) (Figure 4).

#### 3.2.2. BoP Agreement by Educational Level

**Figure 5:**
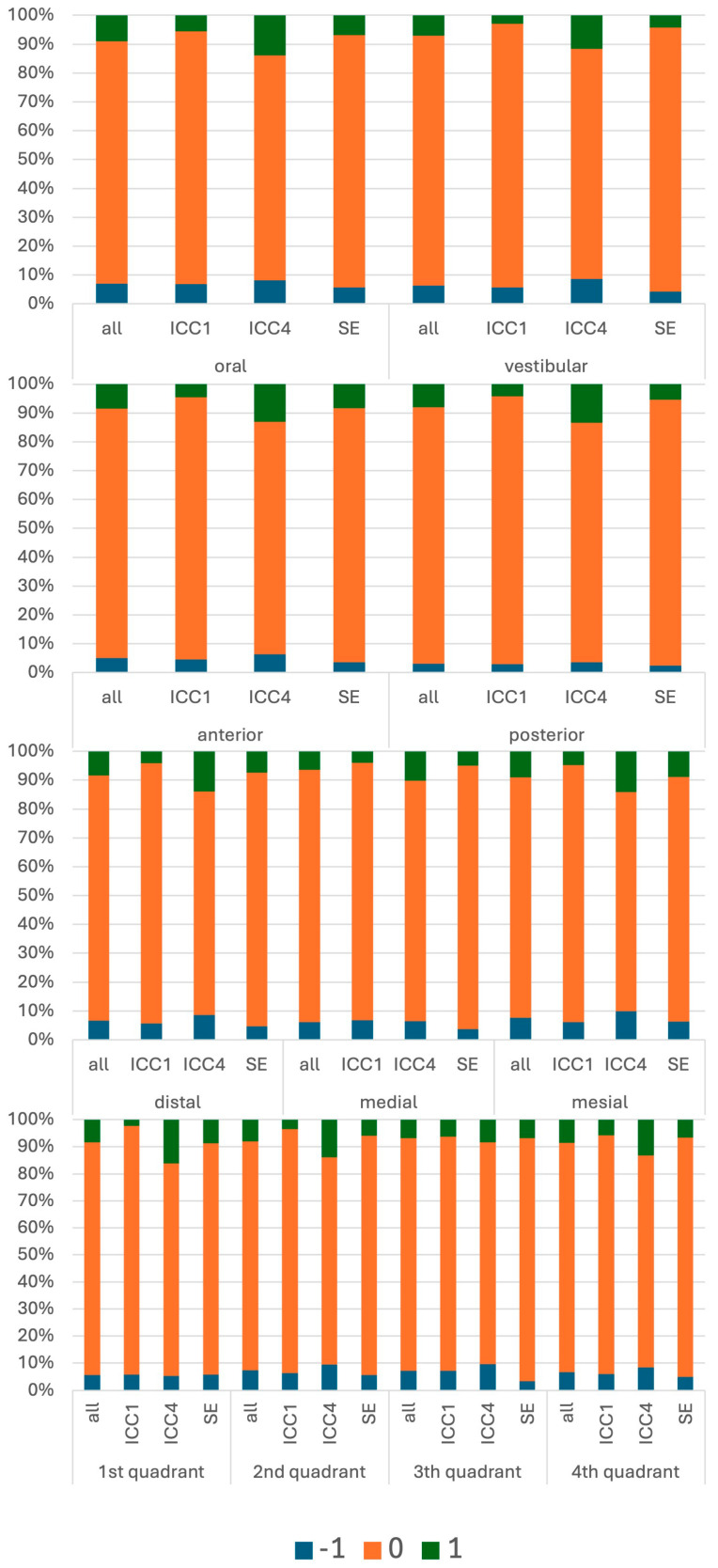
Percentage distributions of measurement differences in bleeding on probing (BoP), analyzed relative to students’ level of knowledge (ICC1: first clinical course; ICC4: advanced clinical course; SE: state examination) and by location. Negative values (−1) indicate underestimation by the student relative to the educator recording, 0 indicates agreement, and positive values (+1) indicate overestimation.

Agreement differed significantly between educational cohorts (global χ^2^ = 52.7; *p* < 10^−6^; Cramér’s V = 0.07). In ICC1, agreement with educator reference values was 85.5%; under-ratings occurred in 9.1% and over-ratings in 5.4% of sites. In ICC4, agreement was 83.4%, with fewer under-ratings (5.6%) but an increased proportion of over-ratings (11.0%). In the state examination (SE), agreement was highest (87.4%), with under-ratings in 6.9% and over-ratings in 5.7%. Thus, the error profile shifted from predominantly underestimation in early clinical training to more frequent overestimation in the advanced clinical course, whereas SE recordings showed the highest overall agreement (Figure 5).

**Figure 6:**
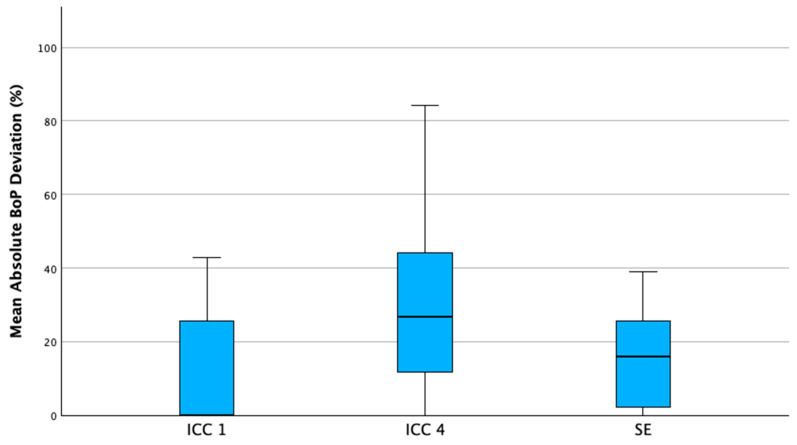
Distribution of patient-level mean absolute bleeding on probing (BoP) deviations (%) across educational levels (ICC1: first clinical course; ICC4: advanced clinical course; SE: state examination). Boxplots illustrate median, interquartile range, and variability of individual patient-level deviations.

Boxplot analysis of patient-level mean absolute deviations revealed distinct variability patterns across educational levels (Figure 6). In addition to site-level analyses, patient-level mean absolute deviations were evaluated for both BoP and tooth mobility to illustrate variability in disagreement patterns between individuals. BoP deviations showed greater dispersion in ICC4 compared with ICC1 and the state examination, indicating increased variability during intermediate stages of clinical training.

#### 3.2.3. BoP Agreement by Periodontal Severity

**Figure 7:**
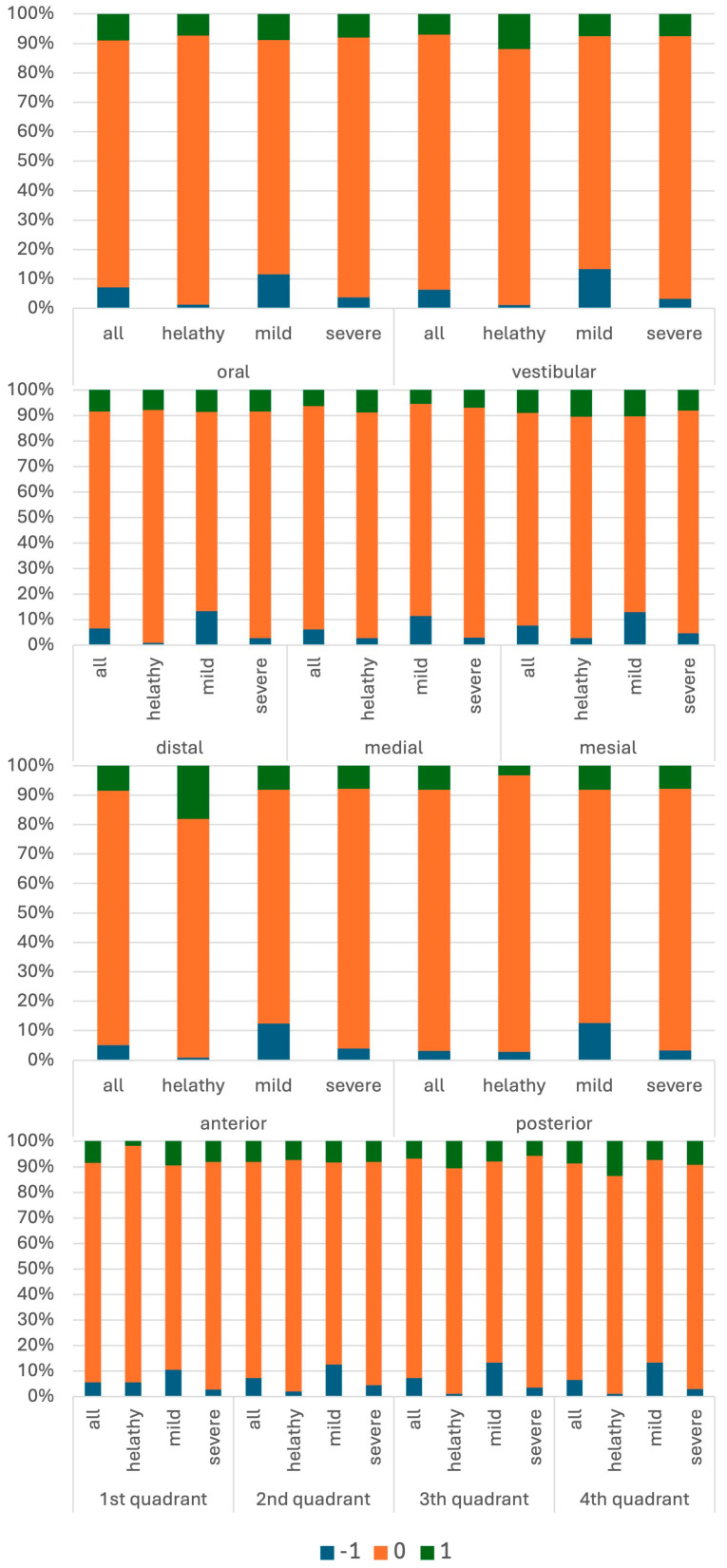
Percentage distributions of measurement differences in bleeding on probing (BoP) relative to severity and location. Negative values (−1) indicate underestimation by the student relative to the educator recording, 0 indicates agreement, and positive values (+1) indicate overestimation.

BoP disagreement was significantly associated with periodontal severity (global χ^2^ = 29.1; *p* < 0.001; Cramér’s V = 0.08). In clinically healthy conditions (*n* = 342), agreement exceeded 91%, with both under- and over-ratings occurring in <5% of sites. In mild disease (*n* = 2910), agreement was 79.6%, with 11.6% over-ratings and 8.8% under-ratings. In severe periodontitis (*n* = 4608), agreement was 88.2%, while 8.1% of sites were over-rated and 3.7% under-rated (Figure 7).

#### 3.2.4. BoP Agreement by Anatomical Location

BoP disagreement patterns differed significantly by anatomical site (all sub-comparisons *p* < 0.001). Oral sites were more frequently under-rated, whereas vestibular sites were more frequently over-rated (χ^2^ = 18.4; *p* < 0.001; Cramér’s V = 0.04). Interproximal site position (distal/mesial vs. mid-sites) and quadrant also influenced disagreement patterns (χ^2^ = 46.9 and χ^2^ = 40.2, respectively; *p* < 0.001), although effect sizes were small (V ≈ 0.04–0.05). The strongest location effect was found for anterior vs. posterior sites (χ^2^ = 165.8; *p* < 10^−12^; Cramér’s V = 0.21), with substantially higher over-rating rates in anterior regions. These findings suggest that anatomical and perceptual factors may contribute to systematic differences in BoP recording accuracy.

#### 3.2.5. Associations of BoP Disagreement with Probing Depth Deviation and Patient Age

**Figure 8:**
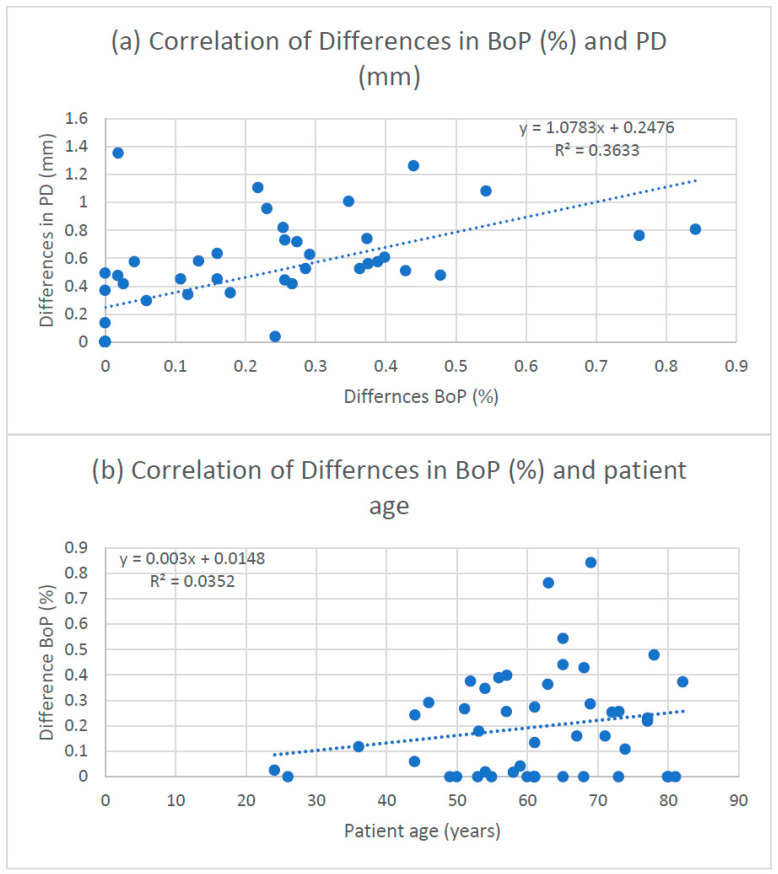
Correlation between measurement deviations of the bleeding on probing (BoP) and (**a**) measurement deviations of probing depth (PD) and (**b**) patient age.

Mean BoP deviations correlated with mean probing depth deviations (r = 0.60; adjusted R^2^ = 0.35; Figure 8a). Detailed probing depth accuracy results have been reported previously [24]. A weak positive association was observed between BoP deviation magnitude and patient age (r = 0.187; Figure 8b).

### 3.3. Mobility

#### 3.3.1. Distribution of Mobility Recordings and Overall Student–Educator Agreement

**Figure 9:**
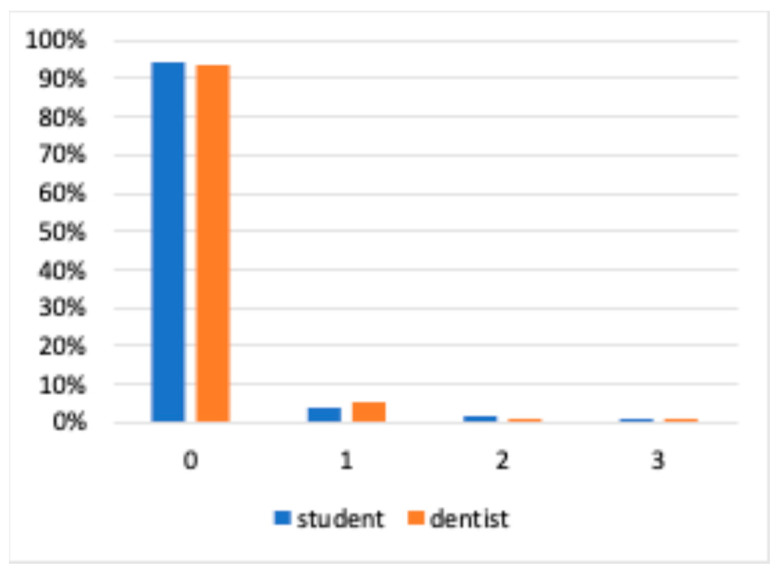
The percentage distribution of the degrees of tooth mobility is shown by the cohort of both students and dentists.

The raw distribution of mobility grades was similar between students and educators. In both groups, grade 0 accounted for the clear majority of recordings (>93%), followed by grade 1 in a small proportion of cases; higher grades (2 and 3) were rare (Figure 9).

**Figure 10:**
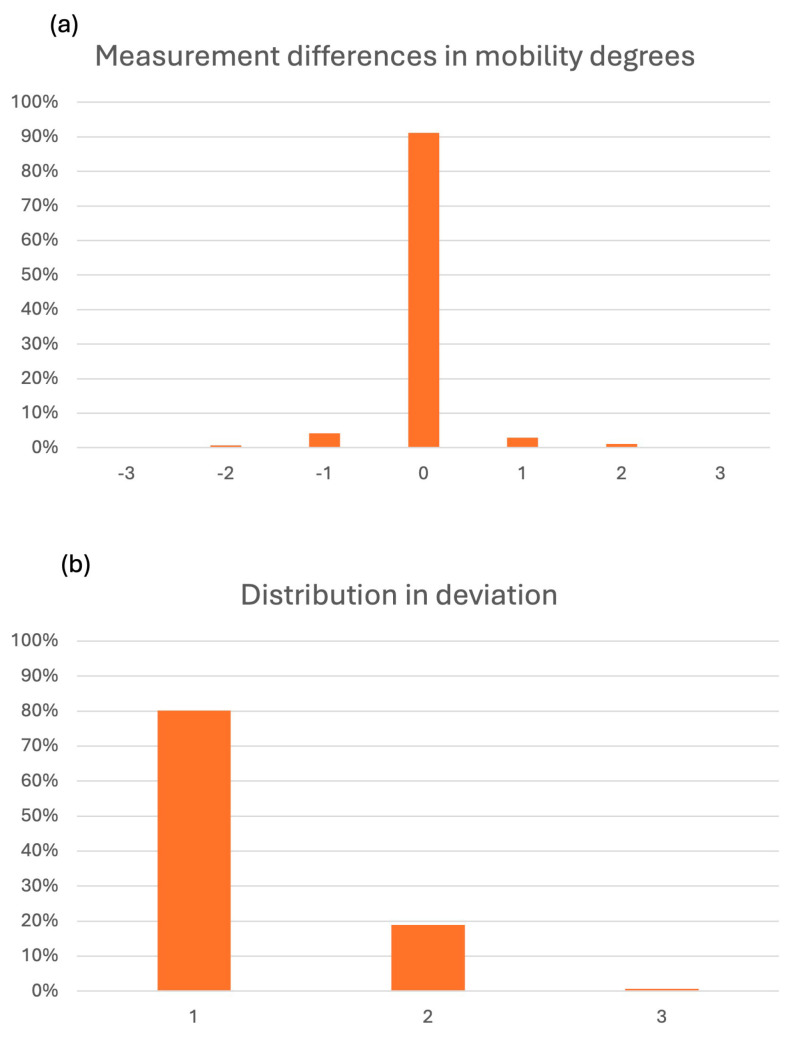
(**a**) Divergences in mobility measurements. Negative values (−3 to −1) indicate underestimation and positive values (+1 to +3) indicate overestimation relative to the educator recording, with increasing absolute values reflecting greater deviation in mobility grading. (**b**) Percentage distribution of divergence magnitudes (0–3), representing the absolute difference in mobility grades between student and educator recordings.

When comparing recordings record-by-record, more than 91% of mobility recordings were identical between students and educators (Figure 10a). Among discrepant recordings, disagreement was predominantly limited to small deviations: the majority differed by ±1 grade and fewer by ±2 grades; ±3 deviations were rare (Figure 10b)**.**

#### 3.3.2. Mobility Agreement by Educational Level

**Figure 11:**
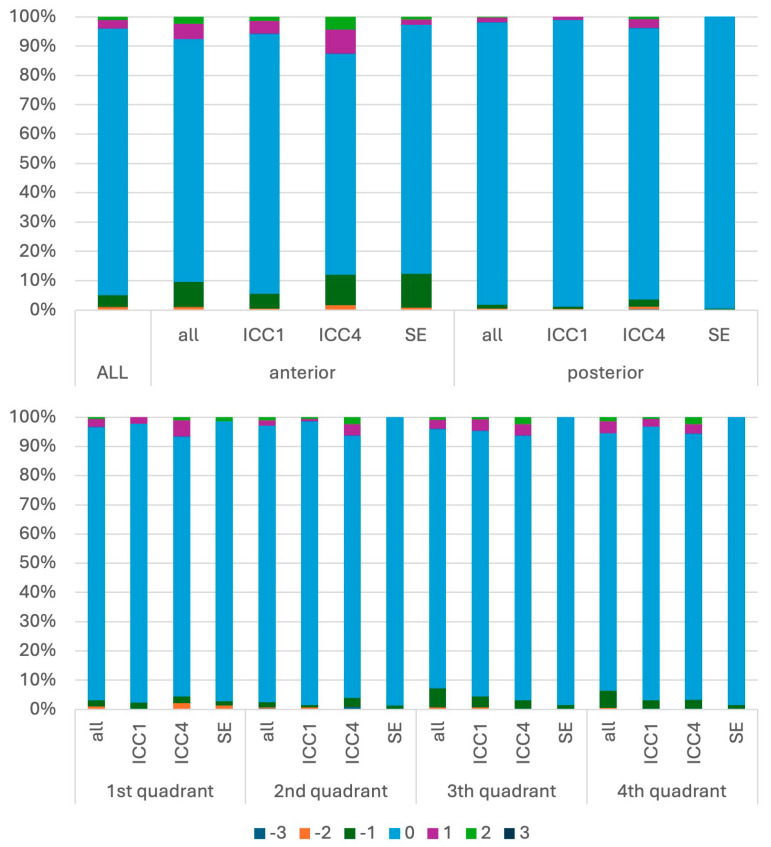
Percentage distributions of mobility measurement differences, analyzed relative to students’ level of knowledge (ICC1: first clinical course; ICC4: advanced clinical course; SE: state examination) and by location. Negative values indicate underestimation and positive values indicate overestimation relative to the educator recording, with increasing absolute values reflecting greater deviation.

Mobility agreement differed significantly by educational level (global χ^2^ = 59.2; *p* < 10^−14^; Cramér’s V = 0.25). In ICC1, agreement was 92.6%, with 4.8% under-ratings and 2.6% over-ratings. In ICC4, agreement decreased to 90.4%, and the proportion of over-ratings increased to 6.2% (under-ratings: 3.4%). In SE, the highest agreement was observed (94.9%) with low rates of both under- (2.7%) and over-ratings (2.4%). Overall, mobility recording accuracy showed a moderate association with training stage (Cramér’s V = 0.25) (Figure 11).

**Figure 12:**
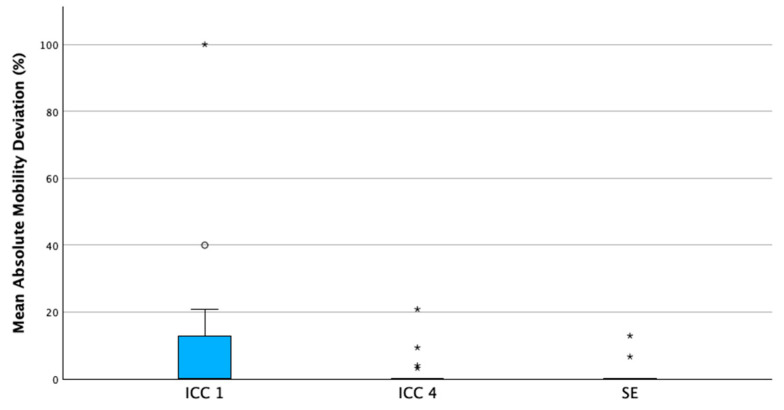
Distribution of patient-level mean absolute mobility deviations across educational levels (ICC1: first clinical course; ICC4: advanced clinical course; SE: state examination). Boxplots illustrate median, interquartile range, and variability of individual patient-level deviations. Mild outliers are shown as circles; extreme outliers are marked with asterisks.

Patient-level mean absolute mobility deviations were predominantly clustered near zero across all educational levels (Figure 12), reflecting the low prevalence of higher mobility grades. Nevertheless, variability and outliers were observable.

#### 3.3.3. Mobility Agreement by Periodontal Severity

**Figure 13:**
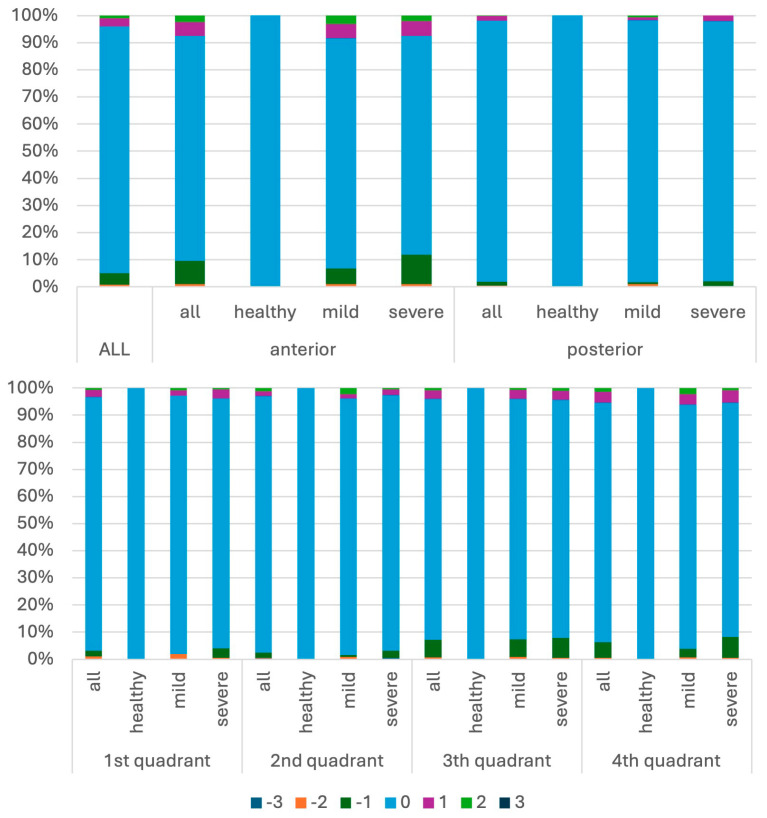
Percentage distributions of mobility measurement differences relative to severity and location. Negative values indicate underestimation and positive values indicate overestimation relative to the educator recording, with increasing absolute values reflecting greater deviation.

Periodontal severity significantly influenced mobility accuracy (global χ^2^ = 41.8; *p* < 0.001; Cramér’s V = 0.18). Agreement was highest in healthy cases (96.4%) and mild cases (94.2%) but decreased in severe periodontitis (89.6%), with higher rates of both under- and over-ratings (Figure 13). This suggests that mobility assessment becomes less consistent with increasing periodontal breakdown.

#### 3.3.4. Mobility Agreement by Anatomical Location

Mobility disagreement differed significantly by anatomical region (all global tests *p* < 0.001). Agreement was lower in anterior teeth (90.6%) than posterior teeth (94.6%) (χ^2^ = 59.2; *p* < 10^−14^; Cramér’s V = 0.25). Quadrant comparisons were also significant (χ^2^ = 36.7; *p* < 0.001; Cramér’s V = 0.12), with agreement ranging from 89.7% (quadrant 1) to 94.0% (quadrant 4).

#### 3.3.5. Association of Mobility Disagreement with Patient Age

**Figure 14:**
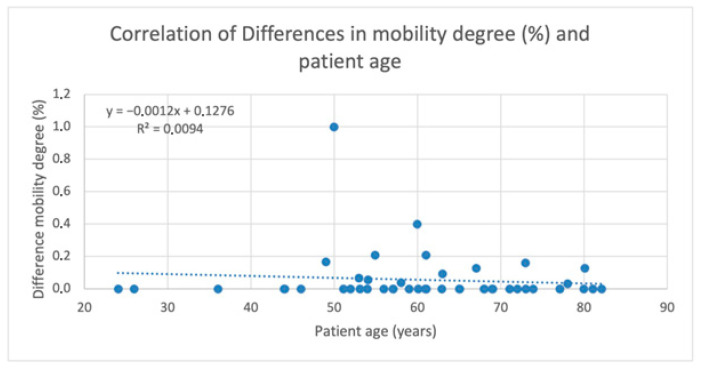
Correlation between measurement deviations of mobility and patient age.

No meaningful association was observed between patient age and mobility disagreement (r = 0.009) (Figure 14). 

## 4. Discussion

### 4.1. Educational Level and Learning Effects

The present study investigated the measurement accuracy of dental students in the collection of the subjective diagnostic parameters of bleeding on probing (BoP) and tooth mobility. The results show that despite a generally very high agreement of the measured values, both the level of training and the localization and severity of the periodontal disease significantly influence the accuracy of the measurements. The findings of the present study partially support the working hypothesis, as systematic disagreement patterns were observed and were associated with educational level, anatomical site, and periodontal severity.

The students’ experience level was associated with the accuracy of both parameters. Students in the first clinical semester (ICC1) tended to underestimate BoP and tooth mobility, whereas students in the advanced clinical course (ICC4) more frequently overestimated these findings. These patterns may reflect differences in examination technique and clinical interpretation across training stages. However, the underlying causes of disagreement cannot be determined with certainty in the present study design. In particular, educator verification represented a repeated examination, and the potential influence of repeated probing on bleeding responses cannot be excluded. Previous studies have demonstrated that probing force can influence BoP responses [10]. However, in the present study, the contribution of probing force to the observed disagreement patterns cannot be isolated [12]. Moreover, BoP has been shown to exhibit high specificity of the absence of bleeding for periodontal stability, whereas the presence of bleeding has limited predictive value for future breakdown [6].

The non-linear learning pattern observed in this study—with a slight decrease in agreement in ICC4 compared with ICC1, followed by the highest agreement in the state examination—suggests that learning progresses with increasing experience, but does not automatically lead to higher diagnostic agreement without continuous calibration and feedback mechanisms. Similar findings have been described in training and calibration studies, in which targeted training significantly improved the agreement between students and experienced examiners [26,27]. These findings support the need to integrate regular calibration units into the clinical curriculum to ensure diagnostic reproducibility.

### 4.2. Subjective Nature of BoP and Mobility

In contrast to previously analysed probing depth outcomes [23], the present findings suggest that learning trajectories differ between metric parameters and more subjective clinical signs. While probing depth measurements mainly reflect millimetre-level technical precision, BoP and mobility additionally depend on tactile perception, probing force control, and interpretive judgement. Furthermore, tooth mobility assessment is inherently examiner-dependent, as the manual evaluation of horizontal tooth displacement relies on tactile perception and clinical judgement. Accordingly, differences between student and educator recordings may partly reflect examiner-related variability. This may explain why error profiles in BoP and mobility show more pronounced directional shifts (under- vs. over-rating) across training stages.

Given the observed influence of probing force on BoP assessment, the use of pressure-calibrated probes could represent a valuable adjunct in undergraduate training. However, such devices are not routinely integrated into the curriculum at our institution. Undergraduate training primarily aims to prepare students for routine clinical practice, where conventional manual periodontal probes remain the standard diagnostic instrument. Consequently, students are deliberately trained to develop tactile sensitivity and force control using standard probes rather than relying on instrument-based force regulation. Nevertheless, pressure-calibrated probes may offer benefits as an additional calibration tool, particularly during preclinical or introductory training phases, to enhance awareness of probing force and improve measurement consistency.

### 4.3. Influence of Periodontal Severity and Clinical Context

The accuracy of student BoP and mobility measurements varied with periodontal severity. This may reflect that bleeding responses are more consistently elicited in advanced inflammation, thereby reducing false-negative recordings in severe cases. While the match rate was over 90% in healthy patients, it decreased significantly in periodontitis cases, with the lowest agreement observed in mild disease. In highly inflamed regions, students tended to overestimate BoP, suggesting increased visual perception and sensitization to bleeding signs. In the case of tooth mobility, a similar mechanism may have contributed to overestimation, presumably due to larger amplitudes of movement and lack of experience in differentiating between physiological and pathological mobility. Expectation bias may also have contributed to the overestimation—due to the probing depths previously collected as part of the systematic periodontal diagnosis, the students should have been aware of the degree of severity. This prior information could have unconsciously influenced the perception and favored a higher probability for the evaluation of a bleeding reaction as “positive”. Such cognitive biases are well documented in diagnostics and can significantly impair the objectivity of clinical findings [28,29,30]. These results are consistent with studies describing higher variability of clinical parameters in inflamed tissues [1,2,12,31].

### 4.4. Association with Probing Depth and Anatomical Factors

Particularly striking was the positive correlation between the deviations of the BoP measurements and the erroneous determinations of the probing depths (PPD). This association suggests that both parameters may be influenced by shared examiner- or site-related factors. However, no causal relationship can be inferred from this observation. A slight but significant association with patient age also suggests that age-related changes in tissue may influence the subjective assessment of bleeding responses. Age-related changes in compliance are also possible. This association should be interpreted in the context of previously published probing depth accuracy data from the same dataset [24].

In line with the observed influence of periodontal severity, the results suggest that diagnostic precision may vary in advanced periodontitis within this study setting, potentially reflecting increased clinical complexity. Although the primary analysis was conducted at the site level, patient-level summaries indicate that disagreement patterns may cluster within individuals and could therefore influence clinical interpretation in selected cases. These findings may be relevant for teaching and could support the use of targeted, experience-based case selection and simulation-based training.

The location of the measuring points had a significant influence on diagnostic accuracy. However, disagreement patterns were not uniform across sites and varied depending on the underlying periodontal severity. Overall, anterior sites showed higher rates of disagreement than posterior sites, and significant differences were also observed between vestibular and oral surfaces. Rather than reflecting a consistent tendency toward over- or underestimation at specific locations, the direction of disagreement appeared to depend on the clinical context, particularly the inflammatory status of the tissue. These findings suggest that, in addition to technical accessibility, visual–tactile perception and contextual interpretation may contribute to measurement variability. Limited visibility and access in posterior regions may increase uncertainty, while visually prominent areas (e.g., anterior teeth) may be more prone to perceptual bias, especially when bleeding signs are subtle. Accordingly, the results support the concept that both anatomical conditions and cognitive perception biases influence the reliability of BoP and mobility recordings [32].

### 4.5. Limitations

This study has several limitations that should be considered when interpreting the findings. The measurements obtained by the dentists serving as the control group were not verified by a single examiner, but by several supervisors from the two respective courses. In future investigations, calibration and verification by one consistent examiner would be advisable to minimize interindividual variation within the control group. As this was a retrospective study, pre-study calibration of the participating dentists was not feasible. Nevertheless, all examiners are actively involved in dental education and regularly conduct periodontal training sessions. However, this does not replace formal study-specific calibration. In addition, parameters such as tooth mobility are inherently examiner-dependent, and the involvement of multiple supervising dentists may have contributed to inter-examiner variability in the reference assessments. This inter-examiner variability may have influenced the observed disagreement patterns. Furthermore, as educator verification was performed after student probing, the potential influence of repeated probing on bleeding responses cannot be excluded. Therefore, the educator recordings should be interpreted as a pragmatic reference within a routine teaching setting, rather than as a fully standardized gold standard.

The present findings make it clear that diagnostic precision does not come from experience alone, but depends to a large extent on systematic training, calibration and standardized procedures. This is especially true for subjective parameters such as BoP and mobility, where even minimal differences in tactile sensitivity or pressure behavior can cause significant measurement deviations. International studies show that intensive calibration programs that combine theoretical units with practical exercises can improve intra-individual accuracy by up to 95% [26,33]. For training, this means the use of training models, feedback systems and objective reference measuring devices (e.g., Periotest^®^) [21,34] can significantly increase reliability. The findings further suggest that strengthening calibration elements within the periodontal curriculum may represent a useful approach. Such measures could further standardize probing force during probing depth measurements and, consequently, BoP assessment. Providing students with access to pressure-calibrated probes could allow regular self-checking of probing force and improve standardization of BoP recording. A combination of repeated hands-on training, structured educator feedback, and supervised exercises in controlled settings may help reduce discrepancies between student and instructor recordings and support confidence in clinical evaluations.

## 5. Conclusions

Within the limitations of this retrospective study conducted in a routine teaching setting, the findings suggest that the diagnostic accuracy of bleeding on probing and tooth mobility assessments may be influenced by examiner experience, anatomical site, and periodontal severity. Despite high overall agreement, systematic disagreement patterns were observed, particularly in anterior regions and in the presence of inflammation, underscoring the potential relevance of standardized calibration procedures. Structured, practice-oriented training combined with repeated feedback mechanisms may contribute to improving the consistency and reliability of clinical findings in undergraduate dental education.

## Data Availability

The original contributions presented in this study are included in the article. Further inquiries can be directed to the corresponding author.

## Abbreviations

The following abbreviations are used in this manuscript:

APTC	Aachen Periodontal Therapy Concept
BoP	Bleeding on Probing
EFP	European Federation of Periodontology
ICC1	Integrated Clinical Course 1 (7th semester; first clinical course)
ICC4	Integrated Clinical Course 4 (10th semester; advanced clinical course)
PMPR	Professional Mechanical Plaque Removal
PD	Probing Depth
PPV	Positive Predictive Value
PSI	Periodontal Screening Index
SD	Standard Deviation
SE	State Examination
SRP	Scaling and Root Planing
WMA	World Medical Association
